# Bilateral Adrenal Incidentalomas: A Rare Presentation of Lung Cancer

**DOI:** 10.1155/2015/179472

**Published:** 2015-05-26

**Authors:** Halit Diri, Melih Kiziltepe, Sulbiye Karaburgu, Mehmet Sait Koc, Ersin Ozaslan, Fatih Tanriverdi

**Affiliations:** ^1^Division of Endocrinology, Erciyes University Medical School, 38039 Kayseri, Turkey; ^2^Department of Internal Diseases, Erciyes University Medical School, 38039 Kayseri, Turkey; ^3^Division of Medical Oncology, Erciyes University Medical School, 38039 Kayseri, Turkey

## Abstract

Adrenal incidentalomas are found incidentally during a radiologic examination performed for indications other than an adrenal disease, and 15% of them are bilateral adrenal masses. This study describes a 51-year-old male smoker patient admitted with diabetes mellitus. An abdominal ultrasonography performed due to his anemia revealed bilateral adrenal masses. His chest X-ray showed abnormal 10 cm opacity at the right upper lung, and brain, thorax, and abdomen CT scans showed multiple lesions compatible with lung cancer metastases. The pathological examination of the transthoracic lung biopsy specimen was consistent with lung adenocarcinoma. Findings in this patient indicate that, in middle aged patients with bilateral adrenal mass and a history or finding of any malignancy, the first diagnosis which should be considered is adrenal metastasis, and confirming the diagnosis by adrenal biopsy may be useless. Furthermore, screening all smoking patients by chest X-ray or thoracic CT for lung cancer may not be accepted as a routine procedure, but in smokers admitted to a hospital due to signs and symptoms attributed to a pulmonary disease, at least a chest X-ray should be requested.

## 1. Introduction

Adrenal incidentalomas are ≥1 cm adrenal masses which are found incidentally during a radiologic examination performed for indications other than an adrenal disease [1]. The widespread application of magnetic resonance imaging (MRI) and computerized tomography (CT), as well as ultrasonography, has led to an increase in the frequency of adrenal incidentaloma diagnosis.

The diagnostic approach to an adrenal incidentaloma should first involve whether it is benign or malign and whether it is functioning or nonfunctioning, because malign and/or excess functioning adrenal masses generally require surgery [2, 3]. Since more than 90% of adrenal incidentalomas are nonfunctioning, the crucial issue is to understand whether the index incidentaloma is benign or malign [1, 4]. The likelihood of malignity is higher in patients with a history or finding of any malignancy, >4 cm adrenal mass, >1 cm/year increase in diameter, bilateral adrenal masses, hyperintensity on T2-weighted MRI, high standardized uptake values on positron emission tomography, and irregular and unclear margins, calcifications, increased attenuation (>10 Hounsfield units) values, and increased percentage (>50%) of contrast washout on unenhanced adrenal CT [1–7].

Bilateral adrenal masses account for 15% of adrenal incidentalomas [8, 9]. The most frequent causes of bilateral adrenal masses are metastatic carcinomas, congenital adrenal hyperplasias, bilateral cortical adenomas, and systemic infiltrative diseases [2]. Herein, we describe a male patient who was incidentally found to have bilateral adrenal mass and diagnosed with adrenal metastasis from lung cancer, by a careful workup.

## 2. Case

A 51-year-old male patient was admitted to another hospital for a routine control of diabetes mellitus which was diagnosed 2 years ago. Although his hemoglobin-A1c and biochemistry tests of serum glucose, urea, creatinine, alanine transaminase, aspartate transaminase, and electrolytes were normal, complete blood count revealed an anemia (hemoglobin: 8.1 g/dL, normal: 14–18 g/dL in males). In order to understand the cause of anemia, he had undergone proctosigmoidoscopy which had revealed external hemorrhoid and an abdominal ultrasonography. Since a 13 cm mass on the left adrenal gland and 7 cm mass on the right adrenal gland with multiple para-aortic lymphadenopathies were found on the ultrasonography, he was referred to Division of Endocrinology of Erciyes University Medical School.

**Figure 1:**
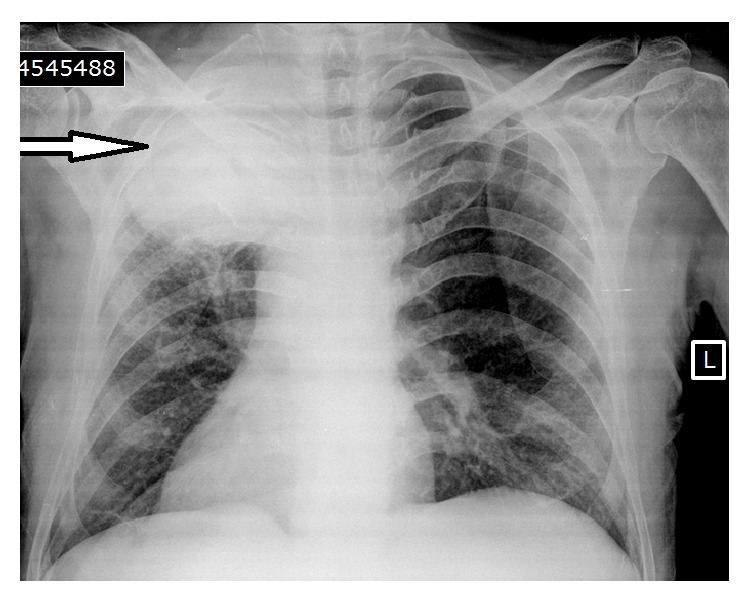
Chest X-ray image showing situs inversus and an abnormal 10 cm opacity at the right upper lung.

The patient reported situs inversus totalis, 39 pack years of smoking, cough, 8 kg weight loss, and diabetes mellitus in his past medical history. A physical examination showed a blood pressure of 110/70 mmHg, heart sounds on the right side of thorax, and clubbing of the fingers. Hormonal analyses showed a baseline serum cortisol concentration of 23.3 *μ*g/dL (normal: 5–25 *μ*g/dL), and his peak cortisol response to 1 mcg adrenocorticotropic hormone (ACTH) stimulation test was also sufficient (19.5 *μ*g/dL). Therefore, an adrenal insufficiency due to bilateral huge adrenal masses was ruled out. His urinary metanephrine and normetanephrine levels were 115 *μ*g/day (normal: <320) and 147 *μ*g/day (normal: <390), respectively. Blood glucose levels monitored by a glucose meter were normal by the treatment of metformin 1000 mg twice daily. Findings on his chest X-ray were abnormal 10 cm opacity at the right upper lung and location of the heart at right side due to situs inversus (Figure 1).

**Figure 2:**
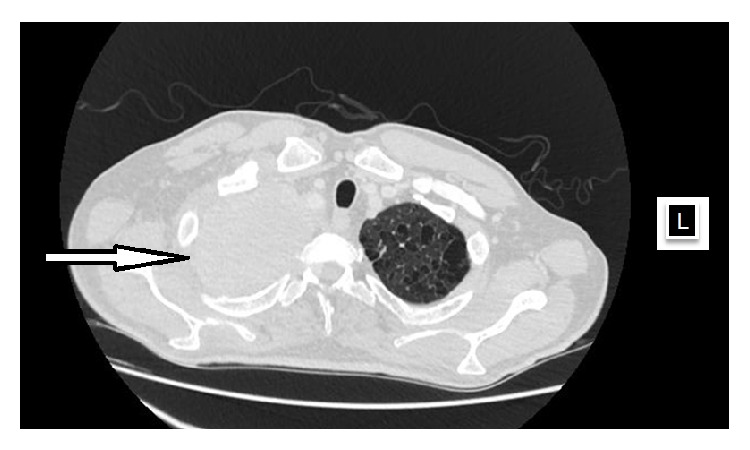
Thorax CT image showing situs inversus and an 11 cm mass opacity at the apex of right lung.

**Figure 3:**
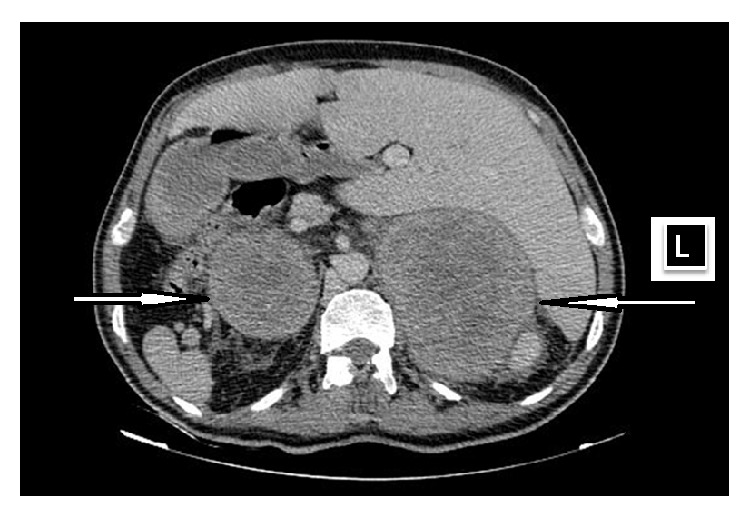
Abdomen CT image showing situs inversus and a 13 cm mass on the left adrenal gland and 7 cm mass on the right adrenal gland.

**Figure 4:**
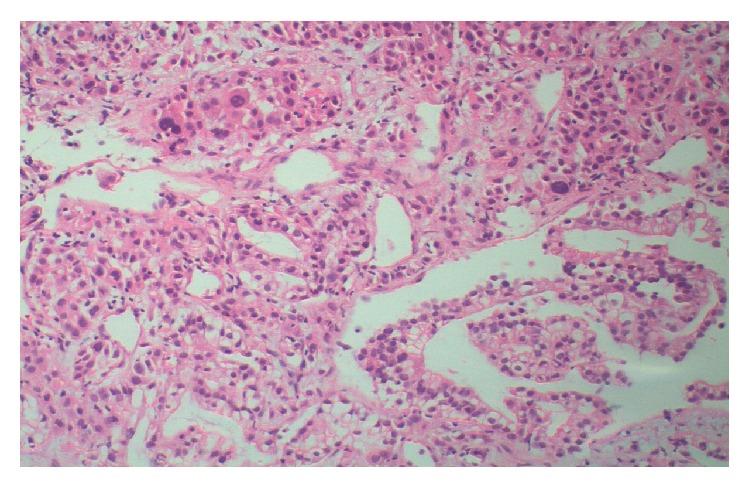
Photomicrograph demonstrating lung adenocarcinoma cells with hyperchromatic nuclei and eosinophilic cytoplasm.

Taken into consideration together, patient age, history of smoking, cough, clubbing, bilateral adrenal masses, multiple para-aortic lymphadenopathies, and abnormal chest X-ray were indicating a metastatic disease. Hence, thorax and abdomen CT examinations were performed, and an 11 cm mass opacity at the apex of right lung, multiple mediastinal necrotic lymphadenopathies, a 13 cm mass on the left adrenal gland and 7 cm mass on the right adrenal gland, multiple para-aortic lymphadenopathies, multiple lytic lesions in thoracic vertebra, and situs inversus totalis were noted (Figures 2 and 3). The adrenal masses had an increased attenuation (42 Hounsfield units) and heterogeneous appearance. In addition, brain CT revealed a 2 cm right parietal lesion. The patient thereafter consulted with Department of Pulmonary Diseases, and a transthoracic needle biopsy was performed. The pathological examination of the biopsy specimen was consistent with lung adenocarcinoma (Figure 4). As a result, the diagnosis of stage IV lung carcinoma was established by the Division of Medical Oncology and thus the patient has undergone chemotherapy.

## 3. Discussion

To find the underlying aetiology of an incidental bilateral adrenal mass, a carefully taken patient history, physical examination, blood tests, and imaging studies should be evaluated together. In patients with multiple metastases like the current case, generally further complicated studies or interventional workup, such as adrenal biopsy, may not be essential. Although CT-guided percutaneous needle biopsy of an adrenal mass is a useful diagnostic method in most patients [10], it may be needless in a patient with multiple metastases. The reasons for that are, firstly, three-fourths of adrenal masses among patients with cancer are metastasis from the primary cancer and secondly the diagnosis of adrenal metastasis will not change the treatment strategy in patients with multiple metastases [2, 3, 11, 12]. Furthermore adrenal biopsy has been shown to lead to adverse events, such as adrenal and liver hematoma, hematuria, pancreatitis, pneumothorax, and tumor seeding along needle track, in 2.8 to 14% of patients [13, 14]. In our case, findings of lung cancer, bilateralism, great size (13 and 7 cm in diameter at left and right side, resp.), increased attenuation and heterogeneous appearance of adrenal masses, multiple metastases on thorax, and abdominal CT scans were enough evidence for the diagnosis of adrenal metastasis from lung cancer.

An important complication of adrenal metastases is intratumoral hemorrhage, which may require adrenalectomy [15]. In the view of endocrinology, hormonal status is the other important issue in a patient with newly diagnosed adrenal incidentaloma. A 24-hour urinary specimen for measurement of catecholamines, 1 mg overnight dexamethasone suppression test, and if patient is hypertensive, a ratio of serum aldosterone concentration/plasma renin activity are routinely used to understand the functionality of the adrenal incidentaloma [2, 11]. However, in the present case, the most important hormonal analyses were levels of baseline serum cortisol and urinary catecholamines, because recognizing adrenocortical hypofunction and bilateral pheochromocytomas is crucial in patients with bilateral adrenal masses. Either pheochromocytoma or adrenocortical hypofunction may lead to their own crisis in patients who are not treated with related medicines before interventional workup or surgeries. Of note, it has been suggested to treat patients with imaging features of pheochromocytoma by *α*- and *β*-adrenergic blockage before interventional operations, even when the results of urinary catecholamine levels are normal [2, 16].

As the size of lung cancer was great and there were multiple metastases, it can be considered that there should be a diagnostic delay in our patient. Although he was admitted to outside hospitals due to smoking and coughs several times, a chest X-ray was not recommended; and therefore lung cancer was overlooked. For smoking patients, particularly who have a family history of lung cancer, routine screening by chest X-ray or thoracic CT has been suggested to diagnose lung cancers early [17–19]. However, it was reported that routine screening for lung cancer might have some risks, such as development of cancer due to radiation involved in CT screenings, useless biopsies and surgeries performed in benign lung lesions, and psychological problems developed due to fear of cancer [20–22].

In conclusion, in middle aged patients with bilateral adrenal mass and a history or finding of any malignancy, the first diagnosis which should be considered is metastasis, and confirming the diagnosis by adrenal biopsy may be useless. Additionally, in a patient with newly diagnosed bilateral adrenal mass, hormonal assays of pheochromocytoma and adrenocortical hypofunction should be performed firstly. Furthermore, screening all smoking patients by chest X-ray or thoracic CT for lung cancer may not be accepted as a routine procedure, but in smokers admitted to a hospital due to signs and symptoms attributed to a pulmonary disease, at least a chest X-ray should be requested. As in the presented case, patients newly diagnosed with bilateral adrenal masses have to be managed by experienced teams of related departments, particularly including endocrinology.
